# Temporal and Spatial Features of the Correlation between PM_2.5_ and O_3_ Concentrations in China

**DOI:** 10.3390/ijerph16234824

**Published:** 2019-11-30

**Authors:** Jiajia Chen, Huanfeng Shen, Tongwen Li, Xiaolin Peng, Hairong Cheng, Chenyan Ma

**Affiliations:** 1 School of Resource and Environmental Sciences, Wuhan University, Wuhan 430079, China; evechen@whu.edu.cn (J.C.); litw@whu.edu.cn (T.L.); chenghr@whu.edu.cn (H.C.); chenyanma@sohu.com (C.M.); 2 Collaborative Innovation Center of Geospatial Technology, Wuhan 430079, China; 3 The Key Laboratory of Geographic Information System, Ministry of Education, Wuhan University, Wuhan 430079, China; 4 School of Geographic Sciences, Xinyang Normal University, Xinyang 464000, China; pxlin2014@whu.edu.cn

**Keywords:** PM_2.5_, O_3_, correlation analysis, spatio-temporal variation, root cause analysis

## Abstract

In recent years, particulate matter of 2.5 µm or less (PM_2.5_) pollution in China has decreased but, at the same time, ozone (O_3_) pollution has become increasingly serious. Due to the different research areas and research periods, the existing analyses of the correlation between PM_2.5_ and O_3_ have reached different conclusions. In order to clarify the relationship between PM_2.5_ and O_3_, this study selected mainland China as the research area, based on the PM_2.5_ and O_3_ concentration data of 1458 air quality monitoring stations, and analyzed the correlation between PM_2.5_ and O_3_ for different time scales and geographic divisions. Moreover, by combining the characteristics of the pollutants, topography, and climatic features of the study area, we attempted to discuss the causes of the spatial and temporal differences of R-PO (the correlation between PM_2.5_ and O_3_). The study found that: (1) R-PO tends to show a positive correlation in summer and a negative correlation in winter, (2) the correlation coefficient of PM_2.5_ and O_3_ is lower in the morning and higher in the afternoon, and (3) R-PO also shows significant spatial differences, including north–south differences and coastland–inland differences.

## 1. Introduction

As one of the largest developing countries, China is seriously affected by air pollution. The Ministry of Ecology and Environment of the People’s Republic of China concluded in the “2016 China Environmental Status Bulletin” that among the 74 major cities in China, the number of days with particulate matter of 2.5 µm or less (PM_2.5_) and ozone (O_3_) as the major pollutants, accounted for 57.5% and 30.8% of the total pollution days in 2016, respectively [1]. Clearly, PM_2.5_ and O_3_ have become the two most serious air pollutants recently.

Both PM_2.5_ and O_3_ could be generated by secondary reaction processes. PM_2.5_ in air usually contains a large amount of toxic and harmful substances and has a long residence time and transport distance in the atmosphere. Therefore, it has become a great burden on human health [2,3,4]. In addition to the primary particulate, the main precursors of PM_2.5_ are oxides of sulfur and nitrogen and volatile organic compounds (VOCs) [5,6,7,8]. O_3_ is a strong oxidizing substance formed by photochemical reaction [9,10]. What is more, ground-level O_3_ is a kind of photochemical pollutant and can cause potential harm to human health [11,12,13,14]. The main precursors of O_3_ are nitrogen oxides (NOx) and VOCs [15,16,17]. Under the action of solar radiation, NOx and VOCs react with oxygen to form O_3_.

In order to cope with the serious air pollution problems, the Chinese government has implemented a series of atmospheric emission control policies in recent years, so that PM_2.5_ concentration has been controlled, to a certain extent. However, the concentration of O_3_ has not decreased, but has in fact increased [18,19]. Guo et al. [20] noted that, after the release of the new environmental protection laws, the concentration of PM_2.5_ decreased by 12.3% between 2013 and 2015. In addition, Li et al. [21] reported that the annual mean concentrations of PM_2.5_, PM_10_, sulfur dioxide (SO_2_), carbon monoxide (CO), and nitrogen dioxide (NO_2_) all decreased from 2014 to 2016 but, at the same time, the annual mean concentration of O_3_ increased by more than 10 µg/m^3^. The above research results show that although the non-attainment rate of PM_2.5_ is still high in most parts of the country [22,23], its concentration has shown a downward trend. However, O_3_, whose concentration has been increasing year on year, has become the major pollutant in most parts of China [21]. As a new mixed pollution model, PM_2.5_ and O_3_ have already caused a huge threat to national health [24,25].

Although the process of air pollutant formation and conversion is very complicated, and the direct relationship between photochemical smog and particulate pollutants is difficult to quantify, it can be seen from the chemical reaction mechanism of PM_2.5_ and O_3_ that they both have common precursors, i.e., VOCs. It is therefore possible that there is a certain relationship between their pollution conditions. Harden [26] analyzed the changes of PM_2.5_ and O_3_ concentrations and their relationship when analyzing the air conditions in the Great Smoky Mountains National Park in the United States and found that these two pollutants showed obvious positive correlations in every season from January 2008 to December 2011. Most scholars that have addressed this issue have taken individual regions of China as the research areas. In the process of analyzing the pollution mechanism of the research areas, the correlation between PM_2.5_ and O_3_ (which we term “R-PO” in this paper) has been determined. The research results showed that, in Beijing (the capital of China, located in North China), the R-PO is positive in summer. In the Changsha-Zhuzhou-Xiangtan district (Hunan province, located in Central China), the R-PO is positive in summer, negative in winter, and is mainly negatively correlated during other periods [27,28]. Chen et al. [29] analyzed the annual correlation between O_3_ and five other types of conventional pollutants (including PM_2.5_) in Jiangsu Province, then found that the correlation differed between the warm and cold seasons. The results showed that the annual R-PO coefficient was less than 0.2, in the hot season it was less than 0.4, and in the cold season it was weak or showed a negative correlation. Wang et al. [30] analyzed the relationship between six conventional pollutants, through dividing China into three regions and treating them as a whole respectively. They found that PM_2.5_ and O_3_ had a strong positive correlation in summer, a negative correlation in winter, and an obvious spatial difference between three regions under the same time. Although the research area was nationwide, the authors did not pay close attention to the correlation between PM_2.5_ and O_3_. In conclusion, PM_2.5_ and O_3_ have different relationships in different regions and during different time periods.

From the above studies investigating the relationship between PM_2.5_ and O_3_, it can be seen that the existing air pollution studies have not regarded R-PO as the research focus. The research related to the relationship between the two pollutants has shown great differences in research areas, time scales, and research methods, so that different conclusions have been drawn under the influence of complex topography, meteorological conditions, and pollutant emissions. In addition, due to the limited space-time characterization of R-PO, there is not enough analytical basis to carry out in-depth analysis and systematic summary of the relationship between the two pollutants. Li et al. [21] speculated that the PM concentrations which displayed a remarkable decrease in most regions, promote the formation of O_3_ because of stronger solar radiation. The simulation results with the Goddard Earth Observing System Chemical Transport Model (GEOS-Chem) by Li et al. [31] indicated that a more important factor for ozone trends in the North China Plain is the ∼40% decrease of PM_2.5_ over the 2013–2017 period, which slow down the aerosol sink of hydroperoxy (HO_2_) radicals and thus stimulate ozone production. Therefore, to obtain a systematical and explicit relationship between PM_2.5_ and O_3_ which is not limited to a certain area, it is necessary to describe the spatial-temporal characteristics of R-PO in detail.

This study is based on PM_2.5_ and O_3_ monitoring data of all the ground monitoring stations in mainland China, with the research period from March 2013 to February 2017. The temporal characteristics and spatial distributions of each stations’ R-PO are described in the daily mean scale and hourly scale respectively, according to the seasonal divisions. Considering the spatial-temporal characteristics of R-PO, the causes of the R-PO patterns are further discussed with regard to the physical properties and chemical mechanisms of the pollutants and the climatic and meteorological factors. Through the analysis process of this paper, we can comprehensively and systematically gain an understanding of the spatial and temporal differences of R-PO and the possible influencing factors from a macroscopic perspective, to provide statistical evidence for the fine-scale study of the chemical mechanisms and physical properties, and the influence of meteorological factors on pollutants. We hope that the results of this study will help people to better understand the relationship between PM_2.5_ and O_3_ and provide a reference for the possible inclusion of this variable in the retrieval models of other parties.

## 2. Materials and Methods

### 2.1. Research Area and Data

The research area of this study—mainland China—is located in the southeast of Eurasia, on the west coast of the Pacific, with a total area of 9.6 million square kilometers. This vast area has complex topography. In addition, the unique geographical location and complex terrain create complex climatic features in mainland China, i.e., the summer is hot and rainy, the winter is cold and less rainy, and the temperature and rainfall vary greatly between the north and the south. This geographical and climatic complexity adds more variability and heterogeneity to China’s air pollution situation.

**Figure 1:**
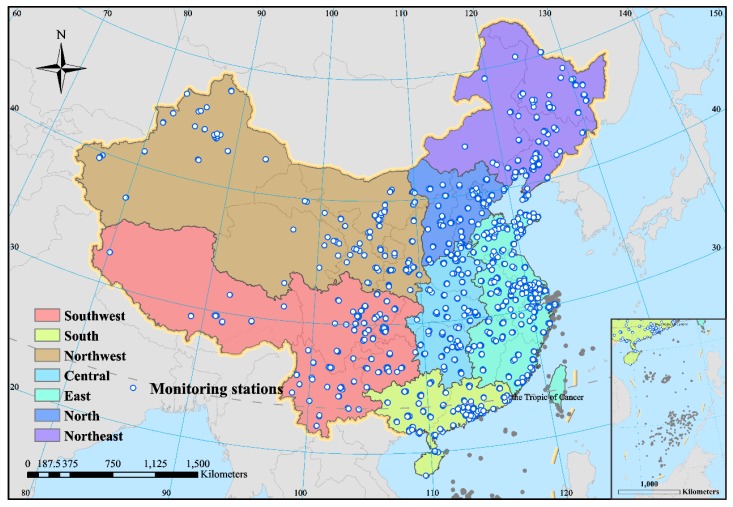
Distribution of mainland China’s ambient air quality monitoring stations.

In this study, we used PM_2.5_ and O_3_ hourly monitoring data with a high temporal resolution for the mainland of China (except for Taiwan province and Hong Kong and Macao Special Administrative Regions), which were obtained from the National Air Quality Monitoring Network, from 1 March 2013, to 28 February 2017. The spatial distribution of the latest air quality monitoring stations is shown in Figure 1. The data source of this study was the “National Urban Air Quality Real-time Publishing Platform” (http://106.37.208.233:20035/).

Since there are a few abnormalities in the raw observation data, the hourly PM_2.5_ and O_3_ monitoring data were preprocessed. The observation values which were abnormally high or congregated unreasonably were removed (PM_2.5_ ≥ 2000 µg/m^3^, PM_2.5_ = 1000 µg/m^3^, and O_3_ = 1200 µg/m^3^). The preprocessed results showed that the number of effective sites nationwide on 1 March 2013, was 494, distributed in 74 cities, which gradually increased to 1544 by 28 February 2017, distributed in 367 cities across the country, covering all prefecture-level administrative regions in mainland China. It is worth mentioning that, to ensure the reliability of the daily average concentration, the daily average data were used only if the effective hours of the original observation data were greater than 18 h.

China has a vast territory spanning five time zones, but the air monitoring data released by the National Air Quality Monitoring Network uses unified Beijing time (UTC + 8, UTC: Coordinated Universal Time). In order to get a more accurate diurnal variation, we made local time corrections for each site. The optimization rules are shown in Table 1.

### 2.2. Methods

**Figure 2:**
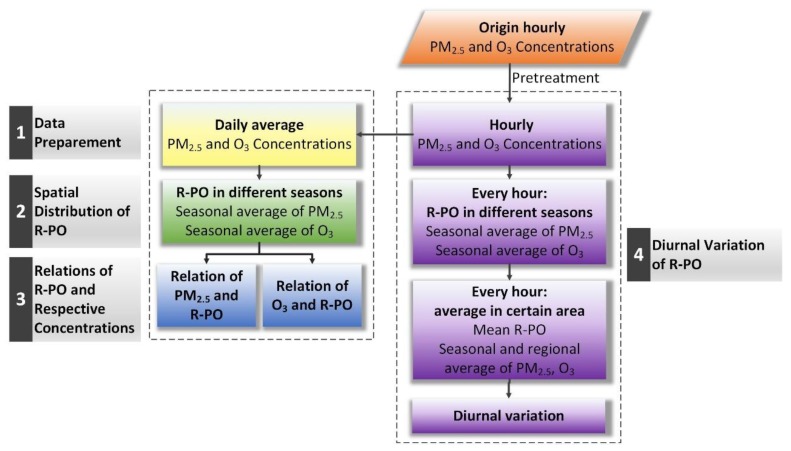
Data organization process. R-PO: the correlation between PM_2.5_ and O_3_.

In order to make full use of the existing data, and to understand the relevance of PM_2.5_ and O_3_, this study was based not only on the PM_2.5_ and O_3_ daily mean concentration at each site, but also the hourly data. Taking the seasonal characteristics of the pollutants into account, the data for each site were divided into season-based units (March–May for spring, June–August for summer, September–November for autumn, and December–February of the following year for winter). In this study, we choose the “Pearson” correlation coefficients to quantify the relevance of PM_2.5_ and O_3_, which was widely used in the research about the relations between different pollutants. The correlation coefficient between PM_2.5_ and O_3_ calculated in Pearson’s way is abbreviated as R-PO. To be specific, R-PO is obtained for each station, one by one, for the PM_2.5_ and O_3_ data pairs in each season over the four years, about 360 days, and the significance level (*p*) values were also calculated at the same time (to distinguish the credibility of R-PO, *p* ≤ 0.05 indicates that the correlation is significant, and *p* > 0.05 means an indistinctive correlation). Accompanied by a *p*-value test, we could get a reliable correlation result of more than 1500 stations. The detailed data organization process is shown in Figure 2.

There are a few points to note: (1) R-PO was first calculated one by one within each site, and (2) when it is necessary to summarize multiple stations, the average processing in the whole country or a certain region was adopted according to the geographical location.

According to the spatial-temporal features of R-PO, the causes of this characterization were then further discussed from two aspects: (1) the influence of the physical properties and the chemical mechanisms of pollutants, and (2) the effect of climatic and meteorological factors on R-PO. For facilitating a clearer and more systematic conclusion, the national situation was also summarized in the research process. Considering the different climatic characteristics and geographical locations of each region, the country was divided into seven regions: Northwest China, Southwest China, Northeast China, North China, East China, Central China, and South China (as shown in Figure 1).

The temporal difference of R-PO is mainly reflected in different seasons and time of day. The spatial heterogeneity is reflected in the differences between different sites or different regions. Overall, in the course of this study, the differences in time and space of R-PO were considered at the same time, and no complete distinction was made.

## 3. Results

### 3.1. Temporal Variation of Particulate Matter of 2.5 µm or Less (PM_2.5_) and Ozone (O_3_) Concentrations

**Figure 3:**
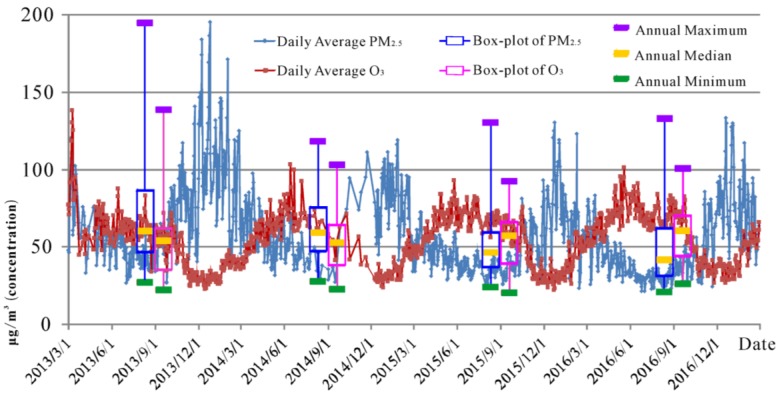
Temporal variation of particulate matter of 2.5 µm or less (PM_2.5_) and ozone (O_3_) concentrations from 1 March 2013, to 28 February 2017.

Change curves of the four-year daily average concentrations of PM_2.5_ and O_3_ and the box plots of their annual mean concentrations are shown in Figure 3, in order to obtain a preliminary understanding of the pollution conditions of PM_2.5_ and O_3_ during the study period from 1 March 2013, to 28 February 2017. It can be seen from Figure 3 that the annual average concentration of PM_2.5_ decreased by 29.8% from 70.4 µg/m^3^ to 49.4 µg/m^3^ over the four years. However, the annual average value of O_3_ concentration increased by 13.7% from 51.6 µg/m^3^ to 58.7 µg/m^3^.

It can be observed from the variation curves in Figure 3 that the concentration changes of PM_2.5_ and O_3_ in each year exhibit a distinct single-peak/single-valley shape. PM_2.5_ concentration is highest in winter and lowest in summer, and O_3_ concentration is highest in summer and lowest in winter. Both of them show significant seasonal differences, and their trends are opposite. The correlation coefficient of this data pair is −0.514, and the significance test *p*-value is less than 0.01, indicating a distinct correlation.

Based on the research of relevant scholars [15,21,32,33,34], PM_2.5_ is normally positively correlated with relative humidity, for which O_3_ is the opposite. Zhang et al. [25] pointed out that the relationship between PM_2.5_ and meteorological factors (including wind speed, sunshine, atmospheric pressure, relative humidity) and the relationship between O_3_ and these factors, are the opposite. Then, in addition to the negative correlation between PM_2.5_ concentration and O_3_ concentration in long time series due to meteorological factors, is there still a deeper connection within each season? In the vast area of China, what kind of spatial heterogeneity will each site show? The analytical studies described in the following sections were conducted so as to further understand the correlation of them.

### 3.2. Spatial and Temporal Differences of R-PO

#### 3.2.1. R-PO of Daily Average Concentrations

**Figure 4:**
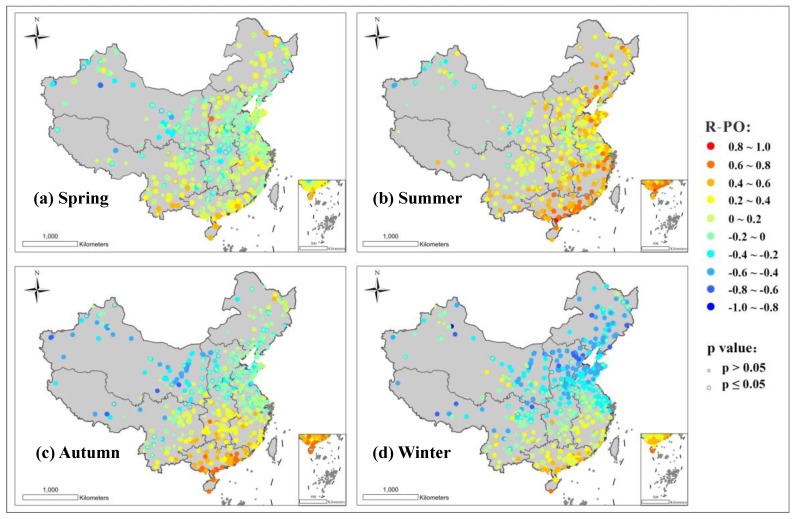
Spatial distribution maps of R-PO in every season: (**a**) spring; (**b**) summer; (**c**) autumn; (**d**) winter.

**Figure 5:**
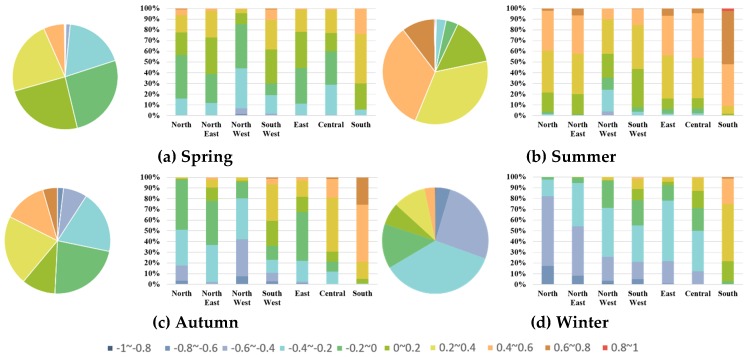
Percentage stacked column charts of R-PO for the different regions in every season: (**a**) spring; (**b**) summer; (**c**) autumn; (**d**) winter.

The spatial distribution of R-PO in the 1458 stations nationwide, in each season, is shown in Figure 4. It can be found from Figure 4 that R-PO has obvious seasonal and spatial differences. In order to more clearly describe the characteristics of the differences, the frequency distribution statistics of the R-PO values passing the significance test were respectively determined. The seasonal and regional differences are shown in Figure 5. Combined with the spatial distribution shown in Figure 4 and the statistical results shown in Figure 5, certain features can be seen. In spring, except for the positive R-PO of the south region (percentage (R-PO ≥ 0.2) ≈ 70%) and the negative R-PO of the northwestern region (percentage (R-PO ≤ −0.2) ≈ 45%), the R-PO of the other regions basically indicates a weak correlation. The summer R-PO is positive, indicating a particularly strong correlation for South China and the eastern coastal areas and a weakly positive correlation for the northwestern region. In autumn, the northwestern region shows a strong negative R-PO (percentage (R-PO ≤ −0.4) ≈ 40%), while there is a strong positive R-PO in South China (percentage (R-PO ≥ −0.4) ≈ 95%), and the Northeast and North China show weak correlation. What is more, the R-PO of winter indicates a negative correlation, on the whole, and most of the northern regions show a high correlation. The above experimental results, showing characterization of each site, are not only consistent with the experimental results of Chen et al. [29] and Wang et al. [30] who all use the method of averaging the concentrations of the monitoring stations in a certain area and then obtaining a synoptic R-PO, as mentioned in the introduction, but also contain more complete details. It can be seen that in each season, there is a significant spatial difference of R-PO between air monitoring stations—the R-PO value in the southeastern part is generally higher than that in the northwestern region. Besides, all stations exhibit seasonal variation, and change in a similar trend—in summer, the relationship between PM_2.5_ and O_3_ is biased towards positive and in winter, it tends to be negative.

#### 3.2.2. R-PO of Hourly Concentrations

**Figure 6:**
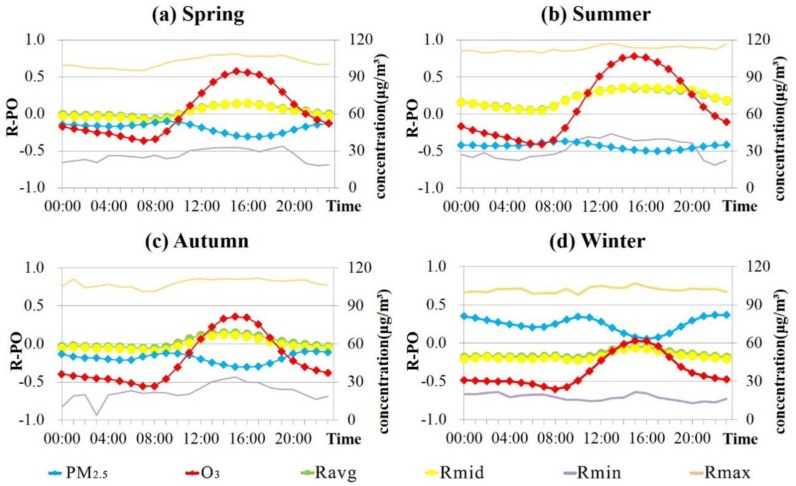
Diurnal variation of PM_2.5_ and O_3_ concentrations and R-PO in every season: (**a**) spring; (**b**) summer; (**c**) autumn; (**d**) winter. (PM_2.5_ represents the mean hourly concentration of all the sites across the country, O_3_ represents the same meaning as PM_2.5_, Ravg is the average of R-PO, Rmid is the median of R-PO, Rmin is the minimum value of R-PO of all the sites nationwide, and Rmax represents the maximum value of R-PO.).

Based on the hourly data, the diurnal change of the PM_2.5_ and O_3_ concentrations across the country and the corresponding R-PO are plotted in Figure 6.

It can be seen from Figure 6 that the variation trends of PM_2.5_, O_3_ concentration, and R-PO in the four seasons are similar, only differing at the numerical level, i.e., there are higher concentrations of O_3_, lower concentrations of PM_2.5_, and higher R-PO values in summer, and there are lower O_3_ concentrations, higher PM_2.5_ concentrations, and lower R-PO values in winter.

Based on the diurnal variation curve, it can be observed that PM_2.5_ peaks at around 10:00, and then gradually decreases, reaching the lowest value of the day around 16:00, and then slowly rises, reaching another peak value at around 22:00, and then maintaining a high concentration at night. The diurnal change of O_3_ concentration is relatively simple compared to PM_2.5_, and is single-peak/single-valley, i.e., the lowest value is around 8:00, gradually rising to around 15:00 when the peak value is reached. It is worth noting that the time of the minimum R-PO (Rmin) value coincides with the peak time of PM_2.5_, i.e., around 10:00, while the time of the maximum R-PO (Rmax) value is consistent with the peak time of O_3_, i.e., around 16:00.

**Figure 7:**
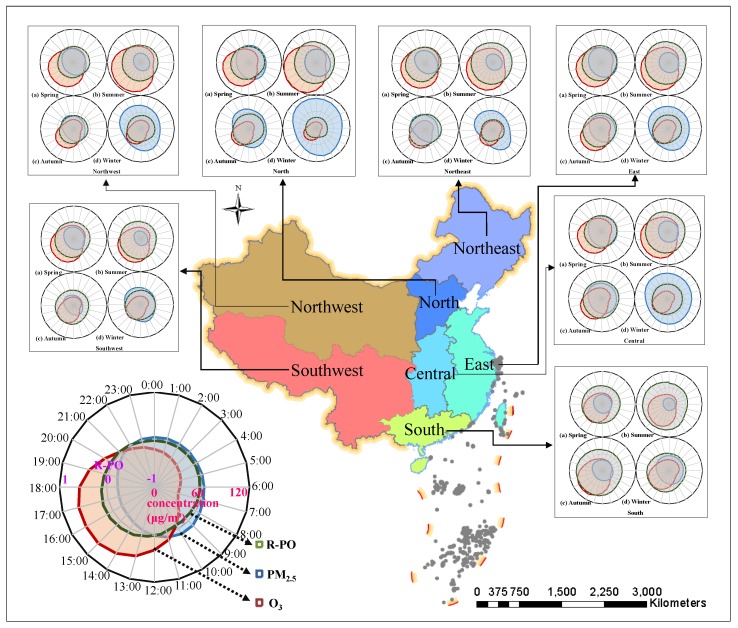
Diurnal variation of PM_2.5_ and O_3_ concentrations and the R-PO of every region, including Southwest, Northwest, North, Northeast, East, Central and South China: (**a**) spring; (**b**) summer; (**c**) autumn; (**d**) winter.

In addition, it can be found from the Rmax and Rmin values in Figure 6 that the regional differences of R-PO are obvious across the country. Since the number of monitoring stations in mainland China is close to 1500, it is difficult to describe them one by one, so the sites are summarized in a partitioned manner. The diurnal changes of R-PO in the seven regions are shown in Figure 7. The results show that the changes in most regions are consistent with the national average. A few areas that are not completely consistent with Figure 6 include the northwestern area in spring, the northeastern and northwestern regions in autumn, and the north, northeast, and northwestern regions in winter. In addition to the characteristics of the previous conclusions (i.e., the lowest R-PO is around 10:00 and the highest R-PO is around 15:00), the R-PO shows obvious increases around 6:00 and 8:00 in the morning, but the fluctuation range is not great.

Comparing Figure 6 and Figure 7 with Figure 3, it can be found that the PM_2.5_ and O_3_ concentrations show opposite trends, not only in the diurnal variation curve, but also in the daily mean concentration curve. By correlating R-PO with the concentration of both pollutants, it can be found that R-PO tends to be positive when O_3_ is higher and PM_2.5_ is lower, and negative when PM_2.5_ is higher and O_3_ is lower.

Therefore, based on the above R-PO patterns in the different seasons and times of the day, it is speculated that the value of R-PO may have a certain relationship with the concentration of PM_2.5_ and O_3_. The physical properties of PM_2.5_ and O_3_ and the chemical mechanisms associated with them may also have an impact on their correlation. In addition, according to the spatial heterogeneity of R-PO in the different seasons and at different times, it can be considered that the climatic differences in the different regions and the meteorological changes over the course of a day may also be related to R-PO. In the following, we provide a cause discussion of the spatio-temporal representation of R-PO from these two aspects.

### 3.3. Cause Discussion of the Spatial and Temporal Differences of R-PO

#### 3.3.1. The Effects of the Physical Properties of PM_2.5_ and O_3_ and the Chemical Mechanisms Associated with Them on R-PO

**Figure 8:**
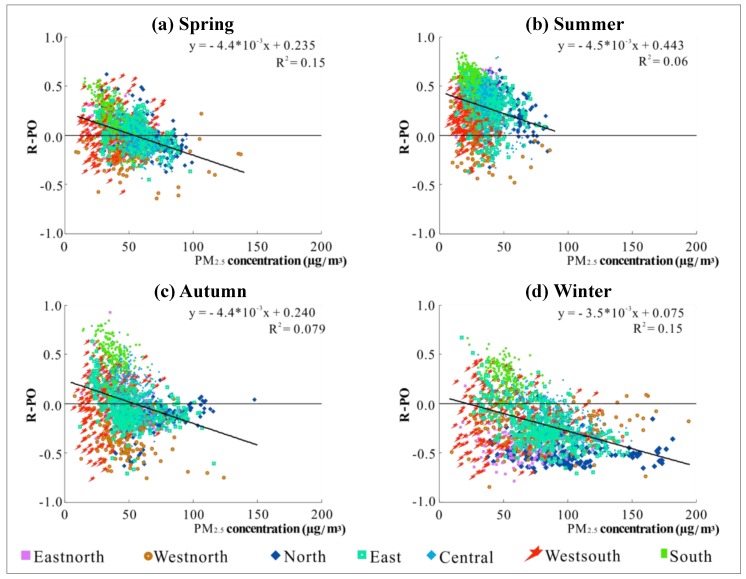
Scatter plots of PM_2.5_ concentration and R-PO based on the daily mean values in different seasons: (**a**) spring; (**b**) summer; (**c**) autumn; (**d**) winter.

In order to further analyze the influencing factors of R-PO, we start with the relationship between R-PO at all the stations and the average concentrations of PM_2.5_ and O_3_ respectively, of the same position, in the corresponding seasons. The experimental results were used to analyze whether the value of R-PO has a certain correlation with the concentration of PM_2.5_ and O_3_, so as to better link the experimental results with the pollution mechanisms. In addition, to clarify the difference in the R-PO values of different regions, a distinction strategy was made between the regions to which the sites belong. It can be observed from Figure 8 that PM_2.5_ concentration and R-PO show different distribution characteristics in the different seasons. In summer, the concentration of PM_2.5_ is lower, and R-PO values are densely distributed in the positive correlation area. The PM_2.5_ concentration in spring and autumn is slightly higher than that in summer, and the distribution of R-PO is more dispersed than in summer. The concentration of PM_2.5_ is higher in most areas in winter, and the R-PO values are more concentrated in the negative correlation area. Observing the distribution of the scatter points in each season, it can be seen that the scatter plots of the four seasons are all distributed in a triangular shape. This means that as the PM_2.5_ concentration becomes higher, the value of R-PO shows a slight decrease. In other words, the higher the PM_2.5_ concentration is, the more the R-PO tends to be negative.

Based on the research findings on the mechanisms of air pollution, it is known that the light extinction coefficient of particulate matter which can absorb light is very high in some areas, at up to 91.9% [35]. As one of the important components of aerosols, the carbon-containing aerosol can also absorb light because of its mixing effect, and its light absorption rate could reach 37.7% of the total absorption rate of the particles [35,36]. All in all, particulate contaminants absorb a lot of light. For PM_2.5_, an aerosol with an aerodynamic diameter of less than 2.5 µm, it can reflect the concentration level of total aerosol in the atmosphere, to a certain extent. Studies have also shown that the PM_2.5_ concentration is strongly positively correlated with the extinction coefficient [37]. Therefore, in the case of a higher concentration of PM_2.5_, meaning a higher aerosol content in the atmosphere, the extinction effect of the particulate matter and the light absorption effect of the carbon-containing aerosol mixing process both have a great influence on the illumination. In addition, light intensity is an indispensable condition for O_3_ generation [38,39]. This can also explain why R-PO is significantly biased toward negative values, with high PM_2.5_ concentrations in winter and around 10:00 of each day.

In summary, it can be generalized that with high PM_2.5_ concentration, the extinction effect caused by the absorption and reflection of light by the particles and the light absorption effect during the internal mixing of the particulate components may together cause the negative R-PO.

**Figure 9:**
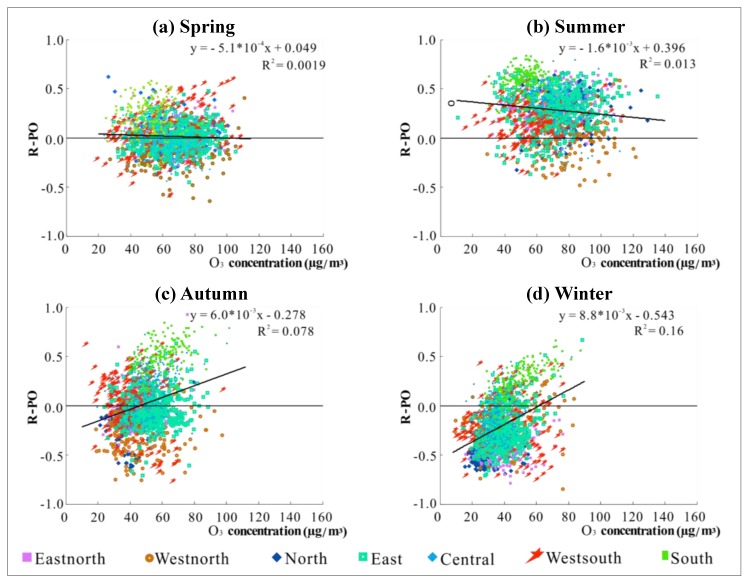
Scatter plots of O_3_ concentration and R-PO based on the daily mean values in different seasons: (**a**) spring; (**b**) summer; (**c**) autumn; (**d**) winter.

Compared with PM_2.5_, the seasonal variation of R-PO with O_3_ concentration is not obvious. Combined with the seasonal characteristics and diurnal variations of R-PO, R-PO tends to be positive when the O_3_ concentration is higher in spring and summer and at 16:00 and is inclined to be negative when the concentration is lower in autumn and winter and around 10:00. Since the change of R-PO with O_3_ concentration is not obvious, it can be speculated that the positive tendency of R-PO may be related to the formation process of O_3_. The regional differences depicted in Figure 8 and Figure 9 are further discussed in Section 3.3.2.

It is known that the proportion of secondary components in PM_2.5_ is very high, and the main components of secondary organic aerosols (SOAs) are sulfate, nitrate, and ammonium salt, formed by condensation and chemical reaction of SO_2_, NOx, NH_3_, and VOCs [40]. The conversions from gaseous pollutant into particulate state and from VOCs into low-volatile and semi-volatile SOAs is affected by light and atmospheric oxidative properties [41,42]. Zhang and Cao [43] noted that the strong photochemical reaction can not only promote the generation of O_3_, but also generate the main components of SOAs at the same time, thus increasing the concentration of PM_2.5_, to a certain extent. Griffin et al. [44] reported that both SOAs and O_3_ are generated by VOCs through photo oxidation and the gaseous/particle equalization process. Shao et al. [45] observed that high-temperature and low-humidity conditions are conducive to the rapid formation of O_3_ and secondary particulate matter, resulting in a composite superposition of high concentrations of O_3_ and PM_2.5_ in the atmosphere. In addition, Guo et al. [46] stated that adequate illumination is especially beneficial for the secondary reaction of PM. Except for the photochemical reaction that simultaneously generates PM and O_3_ resulting in R-PO positive bias, many studies have shown that the oxidation of O_3_ can promote the conversion of primary gaseous pollutants into particulate pollutants. The research of Wonaschütz et al. [42] and Lyu et al. [47] indicated that, in the summer, factors closely related to the formation of new particles include the low wind speed and temperature inversion, as well as O_3_ concentration. This is because the high concentration of O_3_ makes the atmospheric environment highly oxidizing, so that gaseous pollutants such as sulfur dioxide and nitrogen oxide in the air are more easily oxidized and converted into fine particles such as sulfates and nitrates, thereby causing PM_2.5_ concentration to increase.

Based on the above related explanation, it is reasonable to summarize the mechanism as follows. Because the photochemical reaction can generate PM and O_3_ simultaneously, and the oxidation of O_3_ can promote gas/particle conversion, under the combined effect of these two factors, PM_2.5_ and O_3_ possibly tend to show a positive correlation in summer and around 16:00, with the strong light and high temperature. Due to the hysteresis of the secondary reaction, O_3_ usually peaks around 16:00, and the peak of R-PO also appears at 16:00, instead of 14:00, when there is the most intense light. Therefore, it is possible that the promotion effect of O_3_ oxidation influencing PM_2.5_ generation on R-PO may be greater than the effect of the photochemical reaction causing simultaneous generation of PM_2.5_ and O_3_ on R-PO.

#### 3.3.2. The Effect of Climatic and Meteorological Conditions on R-PO

As we all know, there is a high correlation between the climatic characteristics of different regions and the meteorological changes over a short period of time and the concentration of PM_2.5_ and O_3_. Therefore, meteorological factors are bound to affect R-PO by influencing the concentrations of PM_2.5_ and O_3_ under the effect of the physical properties and chemical mechanisms of the pollutants described in Section 3.3.1. In addition, since the R-PO was calculated by four years’ data in this study, the influence of anomalous meteorological changes on the experimental results can be ignored, so that the climatic characteristics of each region in different seasons and the conventional variation characteristics of the meteorological factors were taken into consideration.

Due to the low temperature in the north during winter, the heating of coal leads to an increase of sulfur dioxide and dust particles in the air, which greatly increases the PM_2.5_ pollution. Besides, the low temperature of winter causes the mixed gases from car engines to burn at a slower pace, which leads to incomplete combustion and an increase of motor vehicle exhaust emissions, thus causing an increase of PM_2.5_. Tai et al. [32] and Ji et al. [48] both noted that the low mixed layer height caused by the low wind speed, temperature inversion, and low pressure system in winter hinders the diffusion of primary pollutants and promotes the formation of secondary pollutants and the conversion of gaseous pollutants to a particulate state. What is more, the dry air mass in winter is not conducive to the formation of precipitation, so the rainfall is low, and its duration is short. Furthermore, the wind speed and power are also low, causing the dilution effect on air pollutants to be less obvious, so the concentration of PM_2.5_ is likely to remain high. For O_3_, the high temperature and strong radiation in summer help to increase O_3_ concentration. The effect of PM_2.5_ and O_3_ concentration levels on R-PO is detailed in Section 3.3.1.

In a word, the climatic characteristics of the different regions and the meteorological changes in a short period of time could affect the PM_2.5_ and O_3_ concentration, while PM_2.5_ and O_3_ concentration would make an influence on R-PO (see Section 3.3.1 for details). Other than indirect effects, do the climatic and meteorological conditions have a certain direct impact on R-PO?

Because of the latitude difference caused by the north–south span, the different distances from the sea caused by the east–west span, and the differences in topographical terrain, the different regions of China have various climatic characteristics. Northeast China, North China, East China, Central China, and South China are vulnerable to the monsoons caused by the difference in sea and land temperature, due to their close proximity to the ocean. This is the reason why, for weather characterized by monsoon climate, rainfall and heat usually occur at the same time. The temperature and rainfall gradually increase from the north to the south of China. The northwestern region far from the ocean does not receive many humid air masses, so it is characterized by a dry, less rainy temperate continental climate. In the southwestern region, the high-altitude Qinghai–Tibet Plateau features a plateau/mountain climate with low temperature throughout the year, which is cold and dry with long sunshine duration and strong solar radiation.

Combined with the seasonal variation and the spatial distribution map of R-PO in Figure 3, it can be seen that R-PO shows a significant north–south difference in all four seasons, with the south high and the north low. Furthermore, there is an obvious coastal/inland difference, in that the R-PO is high in coastal areas and low in inland areas, except for the winter, and the positive correlation between coastal areas in summer is extremely obvious. The experimental results show a seasonal feature, in that R-PO has the strongest positive correlation in summer with the strongest light intensity and the strongest negative correlation in winter with the weakest light intensity, as well as a north–south characteristic, in that the R-PO values in the south with strong light intensity are greater than those in the north in all four seasons. Based on the above, it can be considered that strong light intensity may have a close relationship with the positive correlation of R-PO. The experimental results also allow for the conclusion that the photochemical reaction generating O_3_ also promotes PM production, which was described in Section 3.3.1. Therefore, the increase of R-PO in the morning for individual areas in Figure 7 may be due to the relatively dry air in the north and the appearance of early morning solar radiation promoting the positive correlation of R-PO.

What is more, the strongly positive R-PO of coastal areas and the southeast monsoons from the ocean in summer are consistent. As we all know, monsoons from the ocean can bring clean air and abundant precipitation. The cleansing effect of the monsoons may promote the positive correlation of R-PO. Some scholars believed that air masses from the ocean, relatively stable wind speed and wind direction, and strong solar radiation, all facilitate the conversion of gaseous pollutants into new particles [49,50]. According to the above analysis results, the further analysis indicates that the impact of monsoons on R-PO may also be due to the fact that substances such as aerosols in the air can be cleaned by a monsoon, to a certain extent, and thus the solar radiation near the ground is enhanced. Compared with other regions’ R-PO values, the R-PO of the northwest area is negatively correlated in all four seasons, which may be related to the climatic characteristics of inland dryness and less rain. In this case, the pollutants can easily reach the saturation condition, which may suppress the intensity of the pollution formation process, and even cause a certain mutual inhibition between the different secondary reactions, such as the formation processes of PM_2.5_ and O_3_, resulting in the R-PO being biased to a negative value. This is also the reason why Figure 8 and Figure 9 show significant differences between regions with similar PM_2.5_ levels.

Based on the above conclusions, it can be speculated that the diurnal variation of R-PO is also related to the changes of meteorological conditions, such as light intensity, wind speed, relative humidity, and the atmospheric boundary layer. Driven by solar radiation, meteorological conditions show significant changes throughout the day. With the gradual strengthening of solar radiation after sunrise, the wind speed increases with the increase of temperature, and the boundary layer rises. The wind speed always reaches a maximum at around 14:00 and, at the same time, the boundary layer is the highest and the light intensity is the strongest [32,51,52,53,54]. That is why PM_2.5_ and O_3_ always show a positive correlation in the afternoon.

Although the positive value of R-PO is not directly related to the O_3_ concentration, the above analysis results indicate that the main reasons for the high value of R-PO in summer and every afternoon and in coastal and low-latitude areas are strong solar radiation, the cleansing effect of monsoons, and rainfall from the ocean. To find out to what extent the monsoons and rain affect the intensity of light, thereby promoting the positive bias of R-PO, further studies on the interaction between these meteorological factors are needed.

## 4. Conclusions

By using PM_2.5_ and O_3_ concentration data for all the monitoring stations of the China Air Quality Monitoring Network, this paper has presented the spatial and temporal characteristics of the correlation between PM_2.5_ and O_3_. The results show that PM_2.5_ and O_3_ tend to be positively correlated in summer and negatively correlated in winter. In addition, the correlation coefficient between PM_2.5_ and O_3_ is lower at around 10:00, while the value is higher at around 16:00. The correlation between PM_2.5_ and O_3_ also shows obvious spatial differences, including a north–south difference and a coastal–inland difference. Specifically, there is a positive correlation in the south and a negative correlation in the north. Coastal areas tend to be positively correlated, and inland areas tend to be negatively correlated.

According to the spatial and temporal characterizations of R-PO, we can speculate that the level of R-PO is affected by the physical characteristics of the pollutants, the chemical mechanism of air pollution, the climatic differences between regions and seasons, and the meteorological factors in a short period of time. Summarizing all the experimental and analytical conclusions obtained in this paper, we try to speculate the mechanism of R-PO as follows. When humans undertake activities such as industry and travel and emit primary air pollutants, PM_2.5_ and O_3_ are usually produced with the discharge of the primary pollutant, because of their secondary pollutant characteristics. Under this circumstance, the two pollutants usually show a positive correlation. This is why the correlation coefficient of individual regions in the north during spring, autumn, and winter can rise in the morning between 6:00 and 8:00. When the meteorological conditions are more favorable for the formation of PM_2.5_, the two pollutants show a negative correlation, and the higher the PM_2.5_ concentration is, the more strongly it inhibits the formation of O_3_, because of PM_2.5_′s extinction characteristic. When meteorological conditions are more favorable for the generation of O_3_, the two pollutants are positively correlated under the influence of photochemical reaction and atmospheric oxidation. Therefore, it can be postulated that the decrease of PM_2.5_ concentration in recent years has reduced its influence on atmospheric light intensity, thus indirectly promoting the production of O_3_, which demonstrates the simulation result that the decrease of PM_2.5_ concentration has made a certain impact on the O_3_ trend proposed by Li et al. [31]. The mechanism of R-PO affected by chemical and physical factors requires detailed refinement research in small areas. The correlation researches between R-PO and meteorological factors need to introduce adequate potential influencing factors and develop applicable models under the premise that the spatial scale of meteorological data and air monitoring data are consistent. This is the direction that needs further exploration in the future.

However, the phenomenon of O_3_ replacing PM_2.5_ as the primary pollutant in summer is mainly due to the emission conditions of its precursors and the meteorological conditions. Controlling the emission of primary pollutants should be the top priority in the process of environmental air quality control.

## Figures and Tables

**Table 1:** Local time correction table.

Time Zone	Longitude Range	Correction	Number of Stations
UTC + 5	67.5–82.5 °E	[UTC + 8] − 3 h	13
UTC + 6	82.5–97.5 °E	[UTC + 8] − 2 h	46
UTC + 7	97.5–112.5 °E	[UTC + 8] − 1 h	506
UTC + 8	112.5–127.5 °E	-	947
UTC + 9	127.5–132.5 °E	[UTC + 8] + 1 h	32

(UTC: Coordinated Universal Time)
